# Optimization of Cardiac Metabolism in Heart Failure

**DOI:** 10.2174/138161211798357773

**Published:** 2011-12

**Authors:** Tomohisa Nagoshi, Michihiro Yoshimura, Giuseppe M. C Rosano, Gary D Lopaschuk, Seibu Mochizuki

**Affiliations:** 1 Division of Cardiology, Department of Internal Medicine, The Jikei University School of Medicine; 2 Department of Medical Sciences, IRCCS San Raffaele, Roma; 3 Cardiovascular Research Centre, Mazankowski Alberta Heart Institute, The University of Alberta; 4 Musashino University Medical Center

**Keywords:** myocardial glucose and fatty acid metabolism, insulin resistance, metabolic therapy, heart failure.

## Abstract

The derangement of the cardiac energy substrate metabolism plays a key role in the pathogenesis of heart failure. The utilization of non-carbohydrate substrates, such as fatty acids, is the predominant metabolic pathway in the normal heart, because this provides the highest energy yield per molecule of substrate metabolized. In contrast, glucose becomes an important preferential substrate for metabolism and ATP generation under specific pathological conditions, because it can provide greater efficiency in producing high energy products per oxygen consumed compared to fatty acids. Manipulations that shift energy substrate utilization away from fatty acids toward glucose can improve the cardiac function and slow the progression of heart failure. However, insulin resistance, which is highly prevalent in the heart failure population, impedes this adaptive metabolic shift. Therefore, the acceleration of the glucose metabolism, along with the restoration of insulin sensitivity, would be the ideal metabolic therapy for heart failure. This review discusses the therapeutic potential of modifying substrate utilization to optimize cardiac metabolism in heart failure.

## INTRODUCTION

Heart failure is currently a leading cause of death and disability across the globe. Although significant advances in the pharmacological and mechanical (resynchronization therapy, left ventricular assisted devices) treatments have improved the outcome of patients with heart failure, the prognosis of such patients still remains poor. At present, the optimal pharmacological treatment of heart failure targets the suppression of neurohumral activation (such as the renin-angiotensin-aldosterone system (RAAS) and/or β-adrenergic receptor signaling), as well as regulating the fluid volume overload and hemodynamics, and optimizing heart rate. Novel therapeutic strategies acting independently from the neurohumoral axis are required to improve the patient outcomes. Emerging evidence supports the concept that disturbances in myocardial energy substrate metabolism contribute to the progression of cardiac contractile dysfunction and ventricular remodeling in patients with heart failure [1]. 

There is ample evidence to suggest that patients with heart failure have a reduced ability to generate ATP by myocardial oxidative metabolism. Therefore, the optimization of cardiac energy metabolism, without any direct negative hemodynamic effects, is a conceptually attractive therapeutic approach. In this review, we will first outline the normal cardiac energy metabolism, and then focus on the metabolic derangements that occur during heart failure. Finally, we discuss the potential therapeutic applications of optimizing energy metabolism for treating heart failure. 

## MYOCARDIAL SUBSTRATE METABOLISM IN THE NORMAL HEART

**Fig. (1).**
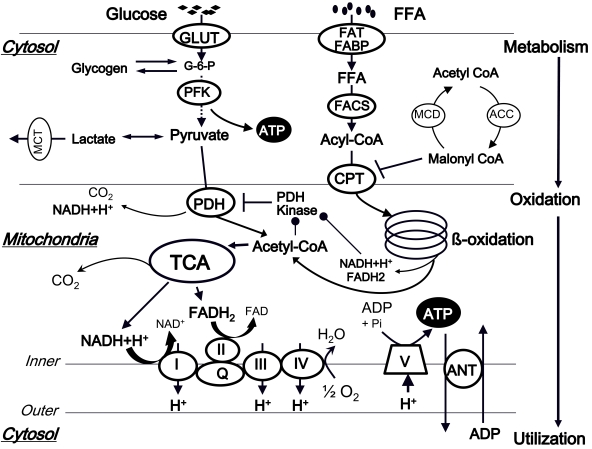
Normal myocardial energy metabolism. ANT, adenine nucleotide translocase; ACC, acetyl-CoA carboxylase; CPT, carnitine palmitoyltransferase; FABP, fatty acid binding protein; FACS, fatty acyl-CoA synthase; FAT, fatty acid transporter; FFA, free fatty acid; GLUT, glucose transporter; G-6-P, glucose-6-phosphate; MCD, malonyl-CoA decarboxylase; PFK, Phosphofructokinase; PDH, pyruvate dehydrogenase; TCA, tricarboxylic acid cycle.

Before the dysfunctional myocardial energy metabolism in heart failure can be fully appreciated, it is important to have a thorough understanding of the regulation of physiological energy metabolism in the normal heart (Fig. **(1**)). 

***The first step: Metabolism***

The utilization of free fatty acids (FFAs) and glucose in the mitochondria accounts for the vast majority of ATP production in the healthy adult heart [2]. Under normal circumstances at rest, 60-90% of the acetyl-CoA which enters the tricarboxylic acid (TCA) cycle comes from the β-oxidation of FFAs, and 10-40% from the oxidation of pyruvate that is derived in almost equal amounts from glycolysis and lactate oxidation [1-4]. However, during conditions of increased metabolic demands, such as increased heart rate or blood pressure, a shift towards a greater utilization of glucose is observed.

### Glucose (Carbohydrate) Metabolism

Glucose transport into cardiomyocytes occurs along a steep concentration gradient and is regulated by the specific transmembrane glucose transporters (GLUTs) in the sarcolemma. Intracellular glucose is phosphorylated to glucose-6-phosphate (G-6-P) by hexokinase, which is then utilized for the glycolytic pathway for energy production and/or glycogen synthesis. Phosphofructokinase (PFK)-1 catalyzes the phosphorylation of fructose-6-phosphate into fructose-1,6-bisphosphate. AMP and fructose 2,6-bisphosphate are positive effectors for this first irreversible step, whereas ATP, citrate, and protons are negative allosteric effectors. Glyceraldehyde-3-phosphate dehydrogenase (GAPDH) catalyzes the conversion of glyceraldehyde 3-phosphate to 1,3-diphosphoglycerate [5], which is ultimately broken down to pyruvate. Pyruvate dehydrogenase (PDH), localized within the inner mitochondrial membrane, catalyzes pyruvate decarboxylation and transformation into acetyl-CoA, which is subsequently fed into the TCA cycle. PDH is inactivated by a specific PDH kinase and is activated by a specific PDH phosphatase. The expression of PDH kinase is increased by starvation, diabetes and peroxisome proliferator activated receptor (PPAR)-α ligands, and the kinase is activated by acetyl-CoA and NADH (produced mainly by fatty acid oxidation (FAO)), which also directly inhibit PDH. In contrast, PDH kinase is inhibited by pyruvate and by decreases in the acetyl-CoA/free CoA and NADH/NAD^+^ ratios. The PDH phosphatase is mainly activated by increased mitochondrial Ca^2+^ entry under such conditions as catecholamine stimulation.

### Fatty Acid Metabolism

The rate of FFA uptake by the heart is determined by the concentration of nonesterified fatty acids (NEFAs) in the plasma. FFAs enter the cardiomyocytes by either passive diffusion or by protein-mediated transport across the sarcolemma, including transport by a fatty acid translocase (FAT) and a plasma membrane fatty acid binding protein (FABP). Once transported across the sarcolemma, NEFAs bind to FABP, and are then activated by esterification to fatty acyl-CoA by fatty acyl-CoA synthetase (FACS). Long-chain fatty acids on fatty acyl-CoA are then transferred to carnitine by carnitine palmitoyltransferase (CPT)-I and the resultant long chain acylcarnitine transported into the mitochondria. The long chain fatty acids from acylcarnitine are then converted back to long chain acyl-CoA by CPT-II. CPT-I plays a key regulatory role in controlling the rate of FFAs uptake by the mitochondria. Malonyl-CoA, which is a potent inhibitor of CPT-I, is produced by acetyl-CoA carboxylase (ACC) and is degraded by malonyl-CoA decarboxylase (MCD). Once in the mitochondria, long chain acyl-CoA undergoes β-oxidation, which releases acetyl-CoA for the TCA cycle, and also regenerates acyl-CoA of two carbons shorter for another round of β-oxidation. The principal products of fatty acid ß-oxidation (FAO) are NADH, FADH_2_, and acetyl-CoA, which generates more NADH and FADH_2_ in the TCA cycle. 

### Interaction of Fatty Acid and Glucose Metabolism

FAO is the primary physiological regulator of glucose oxidation in the heart. High rates of FAO inhibit PDH by PDH kinase activation through an increase in the acetyl-CoA/free CoA and NADH/NAD^+^ ratios (Fig. (**1**)). Conversely, inhibition of FAO increases glycolysis and glucose oxidation both by decreasing citrate levels (which releases inhibition of PFK), and by lowering the acetyl-CoA and/or NADH levels in the mitochondrial matrix, thereby relieving the inhibition of PDH. On the other hand, glucose may also inhibit FAO. Conditions that increase the production of acetyl-CoA from pyruvate stimulate the production of malonyl-CoA, thereby inhibiting CPT activity. 

#### The Second Step: Oxidation

The catabolic pathways for carbohydrates (glucose and lactate), FFAs, and some amino acids all converge on the formation of acetyl-CoA, which serves as the metabolic substrate of the TCA cycle. The TCA cycle has the dual purposes of completing the decarboxylation of acetyl-CoA and of producing NADH and FADH_2_ used in the electron transport chain to provide the energy for ATP synthesis. The flux of the TCA cycle is tightly coupled to the capacity for ATP generation, which depends on the availability of oxygen, the cytosolic ATP/ADP+Pi ratio, and the NADH/ NAD^+^ ratio in the mitochondria. An elevated ATP/ADP ratio under the conditions of low myocardial demand for ATP or the non-availability of oxidized cofactors (NAD^+^ or FAD) is a powerful inhibitor of this cycle. 

The NADH and FADH_2 _derived from glycolysis, FAO, and the TCA cycle are shuttled through the electron transport chain for oxidative phosphorylation. ATP is generated through the sequence of electron transfer to oxygen. 

#### The third Step: ATP Transfer and Utilization

The ATP transfer and utilization for contraction is the final step of the myocardial energetic metabolic loop. The reaction can be expressed as follows: 

Phosphocreatine (PCr) + ADP ↔ Creatine (Cr) + ATP (catalyzed by CK)

This reaction can generate ATP ten times faster than that which occurs *via* oxidative phosphorylation. PCr is a vital energy buffer molecule that provides phosphoryl groups to ADP to rapidly generate ATP. The PCr/ATP ratio is a measure of myocardial energetics, and its reduction may depend on an imbalance of the myocardial oxygen supply and demand. Interestingly, this ratio is reduced in human heart failure [6], thus indicating that this ratio is a significant predictor of mortality [7]. 

The heart has a high energy demand, due to the need for ATP for muscle contraction, and for the maintenance of ATP-dependent cellular processes, including ion transport and intracellular Ca^2+^ homeostasis. Approximately 60-70% of ATP hydrolysis fuels contractile shortening, and the remaining 30-40% is primarily used for sarcoplasmic reticulum Ca^2+^-ATPase (SERCA2A) and other ion pumps (Fig. (**2**)) [1]. 

## MYOCARDIAL SUBSTRATE METABOLISM IN HEART FAILURE

### Energy Production from Various Energy Substrates

Each of the myocardial substrates has a different ATP yield, calculated by the molar value or oxygen equivalents consumed per high energy phosphate produced. FFAs, such as palmitate, provides the highest energy (ATP) yield per molecule of substrate metabolized, mainly through β-oxidation [8]. However, while glucose provides less ATP yield per molecule, glucose metabolism has a greater efficiency in producing high energy phosphates (there is an up to a 40% increase in ATP production per oxygen molecule consumed for glucose versus FFAs) [1, 4, 8, 9]. In other words, FAO requires a greater oxygen consumption for an equivalent amount of ATP synthesized compared to glucose oxidation (approximately 15% more oxygen is required to produce the same amount of ATP from FAO [8, 10]). Moreover, increased FFAs levels, which are frequently associated with heart failure [11, 12], promote the synthesis of uncoupling proteins, which leads to proton leakage, and subsequently, the dissipation of the electrochemical gradient across the inner mitochondrial membrane, resulting in the reduction of cardiac efficiency by limiting ATP production and increasing oxygen consumption [13-15]. Therefore, glucose may become a favorable substrate for energy production in the heart during a state of increased energy metabolic demands, such as heart failure. 

### Significance of Glycolytic ATP Production

Glycolysis contributes only approximately 5% of the total ATP generated in the normally oxygenated heart [10]. During ischemia, the mitochondrial metabolic dysfunction caused by reduced oxygen delivery to the heart results in a decrease in ATP formation by oxidative phosphorylation [4]. The reduction in aerobic ATP formation accelerates glycolysis, glucose uptake and glycogen breakdown, leading to an increase in the contribution of glycolysis as a source of ATP production [2]. During moderate myocardial ischemia, the sustenance of glycolysis may be beneficial. Glycolytically generated ATP is responsible for the maintenance of ion homeostasis, particulary Na^+^/K^+^-ATPase and SERCA2A [16], and thus may be essential for optimal diastolic relaxation [17] (Fig. (**2**)). However, during anoxia or severe ischemia, when glucose oxidation cannot be increased in parallel with the accelerated glycolysis, the increased glycolysis flux results in an accumulation of protons (H^+^) and lactate, which may be detrimental to the heart. Increased Na^+^/H^+^ exchanger activity for the efflux of the accumulated H^+^ during ischemia-reperfusion increases the intracellular Na^+^ concentration, which activates the reverse mode of the Na^+^/Ca^2+^ exchanger and eventually leads to intracellular Ca^2+^ overload [18, 19]. 

### Metabolic Substrate Changes in Heart Failure

Although there is quite a large body of work describing the role of myocardial energy substrate metabolism in the natural history of heart failure, we continue to have a poor understanding of the precise regulatory mechanisms that affect the expression of metabolic proteins. The normal adaptive response of the failing heart involves a complex series of enzymatic shifts and changes in the regulation of transcription factors, ultimately resulting in the switch in substrate metabolism away from FAO toward greater glucose metabolism to maximize efficiency [9, 20]. However, some studies have shown that there is not a decrease in FAO in the early stages of heart failure, and that a dramatic reduction of FAO enzymes, and consequently, the increase in glucose metabolism, occurs only in advanced or end-stage decompensated heart failure [21-23]. In fact, one study using positron emission tomography (PET) demonstrated increased myocardial FFA uptake and reduced glucose uptake in human heart failure [24]. The discrepancies among these clinical investigations may be attributed to the severity of heart failure in the subjects being studied. However, there are also other potential explanations for these results. 

One ptoential mechanism is the presence of "Insulin Resistance" which is highly prevalent in the heart failure population, and plays the pivotal role in the pathogenesis of heart failure [9, 25]. Studies in the canine rapid pacing heart failure model demonstrated a progressive increase in insulin resistance during disease progression [26]. Moreover, elevated plasma FFA levels, which are commonly present in heart failure, impair insulin signaling [27]. Furthermore, the activation of the RAAS occurring in patients with heart failure increases myocardial insulin resistance. In fact, angiotensin II stimulation of cardiac myocytes leads to the inhibition of insulin receptor downstream signaling. Therefore, although glucose is the preferential substrate for metabolism in patients with heart failure (the metabolic shift from FAO to glucose metabolism), insulin resistance inhibits this adaptive response, resulting in further deterioration of heart failure and a state of energy deficiency. Therefore, the ideal metabolic therapy for heart failure would be to induce glucose metabolism, together with the improvement of insulin sensitivity, while simultaneously blocking FFA uptake and FAO. 

### Alternate Considerations for Metabolic Substrate Changes in Heart Failure

There is little evidence of the upregulation of proteins involved in the carbohydrate utilization pathway, even though an increase in glucose metabolism occurs during end-stage heart failure. Furthermore, there is actually either no change (GLUT1 and 4) or downregulation (GAPDH and PDH) of key enzymes involved in carbohydrate metabolism in failing hearts, despite an increase in glucose uptake and oxidation [22, 28]. Recently, a couple reports demonstrated that myocardial lipid storage is reduced in heart failure [29, 30]. Moreover, it has been suggested that glucose oxidation at the mitochondria increases *via* anaplerotic flux in heart failure, thereby compensating for reduced PDH activity and maintaining the TCA cycle flux [31, 32]. Together, these results suggest that any increase in glucose metabolism in heart failure is due to alterations in pathway regulation that are secondary to the suppression of FAO and/or the upregulation of the anaplerotic pathway. However, these shifts indicate a less efficient mode of carbon use for fueling energy synthesis in the myocardium [31]. Therefore, restoration of endogenous fatty acid stores, oxidation and mitochondrial function may be potentially useful as an alternate therapeutic target for heart failure. 

### Alterations in the Expression and Function of Metabolic Regulators and Their Therapeutic Potential for Optimizing Energy Metabolism in Heart Failure

#### AMPK

AMP-activated protein kinase (AMPK) is an important energy-sensing/signaling system, which regulates FAO and glucose uptake in response to altered energy supply and/or demand [33]. This kinase is activated by an increased ratio of AMP to ATP and/or a decreased ratio of PCr to creatine, which then counteracts the increased rates of ATP utilization and maximizes ATP production to meet the energy demands. AMPK stimulates glucose uptake by an increase in GLUT1 expression and GLUT4 translocation to the plasma membrane [34], and accelerates glycolysis by activating PFK-2. The acute activation of AMPK also enhances FAO through a phosphorylation and decrease in acetyl-CoA carboxylase (ACC) activity [33]. In contrast, chronic AMPK activation is associated with decreased expression of CPT-1 and medium chain acetyl-CoA dehydrogenase (MCAD), resulting in decreased FAO [34]. Therefore, AMPK plays an important energy metabolic role through the insulin-independent activation of glucose uptake and glycolysis, with biphasic actions on FAO. Further confirming this role, there is a paper recently demonstrated that macrophage migration inhibitory factor (MIF), which is released in the ischemic heart, stimulates AMPK, and protects the heart against ischemic injury and apoptosis [35].

#### Insulin/IGF-1 Signaling

PI3K-Akt, the down-stream effectors of insulin/IGF-1 signaling, are key regulators of cardiomyocyte growth and survival [36, 37], and are also important modulators of metabolic substrate utilization and cardiomyocyte function [38-40]. It is widely accepted that acute activation or, to be more precise, the acute acceleration (superinduction) of insulin/IGF-1-PI3K-Akt signaling has caridoprotective effects both *in vitro* and *in vivo* [38, 41-44]. In contrast, we and others have previously demonstrated that chronic activation of Akt leads to detrimental outcomes due to either negative feedback inhibition of its upstream molecules, such as insulin receptor substrate (IRS)-1 [45], or a disruption of coordinated hypertrophy and angiogenesis [46, 47]. From the viewpoint of the cardiac metabolism, Akt activation generally promotes the intracellular transport and metabolism of glucose, while it inhibits FFA metabolism [48, 49]. The acute activation of Akt appears to increase glucose uptake, predominantly through enhanced sarcolemmal GLUT4 localization [38], while its chronic activation leads to insulin resistance, with a substantial decrease in GLUT4 both in the intracellular cytosol and on the sarcolemal membrane, in addition to negative feedback inhibition of IRS-1-PI3K coupling [40, 45]. In human advanced heart failure, Akt is paradoxically activated, with the concomitant reduction of IRS-1 [45, 50], thus suggesting a mechanism by which chronic Akt activation may become maladaptive, in contrast to its acute activation (Fig. (**3**)). In contrast, other groups have shown that Akt activity is not elevated in heart failure, or is actually decreased in advanced heart failure due to the disturbed signal transduction of the upstream effectors, including the insulin/IGF-1 receptor, through the IRS-1-PI3K pathway [25, 51, 52]. This discrepancy could be related to the differences in the severity and chronicity of ventricular dysfunction between the studies. Taken together, superinduction of PI3K-Akt signaling appears to play a central role in cardioprotection both *via* modification of energy metabolism and *via* mechanism(s) independent of metabolism, such as the direct anti-apoptotic effects. Likewise, the activation of the insulin/IGF-1 cascade preserves mitochondrial energy metabolism, which in turn counteracts cardiotoxic oxidative stress and promotes survival in heart failure [53]. 

In addition to its metabolic effects, the activation of IGF-1-PI3K-Akt signaling upregulates SERCA2A activity, which leads to enhancement of sarcoplasmic reticulum Ca^2+^ uptake [54-56]. Therefore, the activation of this cascade could represent another therapeutic strategy for the improvement of cardiac contractility in heart failure, although this effect is mostly due to chronic activation of Akt, and would need to be confirmed in additional experimental models. 

#### Pyruvate

Pyruvate increases the contractile function and potentiates the contractile effects of β-adrenergic stimulation in both the normal and failing myocardium [57, 58]. Pyruvate can increase the intracellular Ca^2+^ transients as a result of increased sarcoplasmic reticulum Ca^2+^ accumulation, as well as increased myofilament Ca^2+^ sensitivity as a result of the increased intracellular pH. Similarly, PDH kinase inhibition activates PDH and increases pyruvate oxidation, thus resulting in an increase in mechanical efficiency by switching the heart towards a more efficient fuel. 

#### GLP-1

The elevation of the plasma insulin concentration enhances glucose metabolism and inhibits FAO. Glucagon-like peptide (GLP)-1 is one of the incretins which promotes post-prandial insulin secretion and improves insulin sensitivity. GLP-1 infusion increases glucose uptake and improves cardiac function in a pacing-induced heart failure model [59], although in a human heart failure study, short-term GLP-1 treatment failed to show any beneficial effects [60]. Since GLP-1 is rapidly degraded by dipeptidyl peptidase (DPP)-IV *in vivo*, synthetic GLP-1 analogues with an extended plasma half-life (e.g. liraglutide) and GLP-1 receptor agonists (e.g. exenatide) have been developed. A DPP-IV antagonist would provide an alternative promising agent in this context. Indeed, sitagliptin, one of the DPP-IV inhibitors, has been shown to improve left ventricular performance in response to dobutamine stress in patients with coronary artery disease in an insulin-independent manner [61]. However, at present, there is insufficient evidence to support and/or suggest the use of incretins in heart failure.

#### FFA Metabolism Modulators

Increasing FFA metabolism leads to an increase in oxygen consumption, and is less efficient source of energy compared to glucose metabolism (with regard to ATP production/oxygen consumed), as discussed above. Moreover, if myocardial FFA uptake overwhelms the oxidative capacity of the heart, FFAs can accumulate as intramyocardial lipids (triglycerides, diacylglycerol, long chain acyl CoA’s and ceramides), which are associated with "lipotoxicity", leading to further impairment of the cardiac function, in addition to insulin resistance [3, 27, 62, 63]. In fact, high circulating lipid levels and intracellular accumulation of long-chain fatty acid moieties, such as that which occurs during fasting or in patients with diabetes, enhance PPAR-α mediated expression of PDH kinase, thus resulting in the inhibition of the phosphorylation of PDH. Therefore, pharmacological interventions aimed at lowering circulating FFA levels, inhibiting FFA cellular and/or mitochondrial uptake, and inhibiting fatty acid β-oxidation could provide another therapeutic strategy for optimizing cardiac metabolism. 

Trimetazidine is a metabolic agent initially developed for the treatment of myocardial ischemia. Its effect is achieved by shifting the energy substrate preference from fatty acid oxidation to glucose oxidation, secondary to inhibition of 3-ketoacylCoA thiolase (3-KAT), the final enzyme in β-oxidation [64]. In experimental studies, trimetazidine exerts a cardioprotective effect in* in vitro* models of myocardial ischemia through a rapid restoration of oxidative phosphorylation processes, protection of cardiac cells against the accumulation of protons, and prevention of the intracellular accumulation of sodium and calcium ions. These effects of trimetazidine are believed to help maintain the integrity of cell membranes, as well as maintain mitochondrial structure and function. Because of the preferential promotion of glucose and pyruvate oxidation, trimetazidine improves the activity of two membrane-bound pumps, namely the Na^+^/K^+^-ATPase and SERCA2A, which are responsible for left ventricular systolic and diastolic function, respectively. In clinical studies, trimetazidine treatment leads to a substantial improvement in cardiac function and New York Heart Association function (NYHA) class in the heart failure population [65, 66]. The improvement of the left ventricular function is associated with a reduction of the inflammatory response in patients treated with trimetazidine [67]. Trimetazidine also improves the myocardial PCr/ATP ratio, suggesting that this agent preserves the intracellular levels of myocardial high energy phosphates [68]. 

CPT-1 inhibitors (such as etomoxir, perhexiline and oxfenicine) reduce mitochondrial FFA uptake. As a consequence, myocardial glucose substrate utilization increases [1, 10]. Studies with etomoxir demonstrated that chronic treatment with this agent results in improved sarcoplasmic Ca^2+^ handling and increased SERCA2A expression, leading to improved cardiac function [69, 70]. Unfortunately, this drug can produce serious adverse effects, including liver toxicity. Another recent study has revealed the salutary effects of perhexiline in improving the myocardial oxygen consumption, left ventricular ejection fraction, symptoms, and exercise capacity in maximally treated heart failure patients [71]. 

There are three isoforms of PPARs: PPARα, PPARδ, and PPARγ, all of which are expressed in the heart. While PPARα and PPARδ increase myocardial FAO rates, PPARγ agonists, such as, thiazolidines, actually decrease FAO rates by decreasing circulating FFA levels, and thus decreasing myocardial FFA uptake and oxidation [72].

#### Catecholamines

Short-term stimulation of β-adrenergic receptors increases glucose uptake, glycolysis and glucose oxidation. The activation of protein kinase A (PKA) and Ca^2+^-calmodulin-dependent kinase (CaMK) by epinephrine, an adrenergic receptor agonist, leads to acute increases in PFK and Akt activity [73, 74]. Moreover, epinephrine also increases mitochondrial Ca^2+^ uptake, which activates PDH and other TCA cycle enzymes. In contrast, the long-term upregulation of catecholamines, often present in patients with heart failure, antagonizes the actions of insulin, promotes lipolysis, and increases circulating FFA levels, all of which can lead to insulin resistance [9]. This action is partly mediated through the negative feedback inhibition of either the insulin receptor [74] or of IRS-1 [45]. Adrenergic blockade with carvedilol reduces FFA utilization in favor of greater glucose utilization in heart failure patients [75]. This change in myocardial energetics could provide a potential mechanism for the decreased myocardial oxygen consumption and improved energy efficiency seen with β-adrenergic receptor inhibitors in the treatment of heart failure. 

#### RAAS

Alterations in RAAS is centraly involved in the pathophysiology of heart failure. We and others have previously reported that cardiac ACE activity and gene expression, as well as the local synthesis of angiotensin II and aldosterone, are increased in the failing hearts [76-78]. The persistent activation of these RAAS components contributes to altered insulin/IGF-1 signaling pathways and ROS formation, which induces endothelial dysfunction and insulin resistance [79]. There have been several reports demonstrating that persistent stimulation of aldosterone induces IRS-1 degradation, thus leading to insulin resistance [80, 81]. Moreover, increased aldosterone levels were shown to be associated with insulin resistance in a heart failure population [82]. Therefore, angiotensin-converting enzyme inhibitors, angiotensin receptor blockers, and mineralocorticoid receptor inhibitors may all have favorable effects on the glucose metabolism and thereby restore insulin sensitivity [83, 84]. 

## CONCLUSION

The alterations in myocardial fuel selection and energetics play a key role in the pathogenesis and progression of heart failure. Shifting the energy metabolic pathways away from FFA utilization and toward glucose utilization can be an attractive novel therapeutic strategy for the prevention or early treatment of heart failure in terms of providing a more energy-efficient substrate usage. Meanwhile, special attention should be paid to insulin resistance, which is generally associated with advanced heart failure, and the development of new therapies aimed at the improvement of insulin sensitivity should be considered in order to take advantage of glucose as the preferred metabolic substrate in heart failure. 

## Figures and Tables

**Fig. (2).**
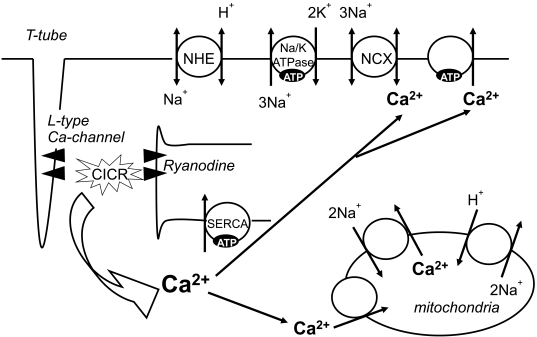
ATP regulates the electrolyte balance, including Ca^2+^ homeostasis. CICR, Ca-induced Ca release; NCX, Na^+^/Ca^2+^ exchanger; NHE, Na^+^/H^+^ exchanger; SERCA, sarcoplasmic reticulum Ca^2+^ ATPase pump.

**Fig. (3).**
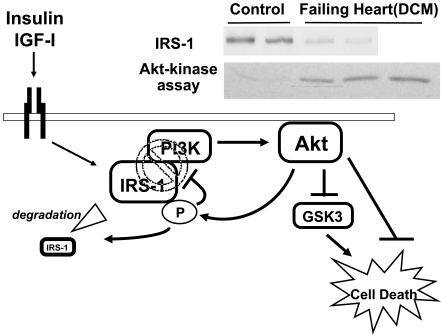
A schematic representation of Akt-mediated feedback inhibition in heart failure. Insulin or IGF-1 signaling exerts cardioprotective effects through the acute activation of its down-stream effectors, such as IRS-1, PI3K and Akt. In contrast, the phosphorylation of IRS-1 induced by chronic Akt activation leads to their dissociation from PI3K, as well as proteasome-dependent degradation, thus leading to detrimental results. Similar negative feedback inhibition of IRS-1 is also observed in human heart failure where there is the persistent activation of Akt. Adapted from reference 45. DCM, dilated cardiomyopathy; GSK-3, glycogen synthase kinase-3; IGF-1, insulin like growth factor-1; IRS-1, insulin receptor substrate-1; PI3K, phosphoinositide 3-kinase
